# Comparison of methods for isolation of extracellular vesicles from bronchoalveolar lavage fluid

**DOI:** 10.20517/evcna.2025.85

**Published:** 2026-04-28

**Authors:** Mark E. Fraser, Radha Patel, Taylor Shinabery, John Zagorski, Ling Chen, Navneet K. Dhillon, Matthias Clauss, Emma H. Doud, Amber L. Mosley, Benjamin Gaston, Homer L. Twigg III

**Affiliations:** ^1^Division of Pulmonary, Critical Care, Sleep, and Occupation Medicine, Indiana University Medical Center, Indianapolis, IN 46202, USA.; ^2^Division of Pulmonary, Critical Care, and Sleep Medicine, The University of Kansas Medical Center, Kansas City, KS 66160, USA.; ^3^Department of Biochemistry, Molecular Biology, and Pharmacology, Indiana University School of Medicine, Indianapolis, IN 46202, USA.; ^4^Center for Proteome Analysis, Indiana University School of Medicine, Indianapolis, IN 46202, USA.; ^5^Center for Computational Biology and Bioinformatics, Indiana University School of Medicine, Indianapolis, IN 46202, USA.

**Keywords:** Extracellular vesicles, bronchoalveolar lavage, size exclusion chromatography

## Abstract

**Aim:** Extracellular vesicles (EVs) have been described and isolated from a variety of biological samples, including bronchoalveolar lavage (BAL). While EVs have been isolated from BAL by various methods, there has not been a comparative study to determine optimal EV isolation methods for this complex and unique biologically derived fluid.

**Methods:** Membrane affinity binding (exoEasy by Qiagen), ultracentrifugation (UC), and size-exclusion chromatography (SEC) EV isolation methods were compared to determine the best utilization of each method when isolating EVs from BAL.

**Results:** Both SEC and UC were able to isolate significant EV populations reliably, with UC yielding higher apparent yields by western blotting and Nanosight particle tracking. However, transmission electron microscopy, total protein assays, and proteomic analysis suggested this was due to free protein and protein aggregate contamination. The exoEasy kit demonstrated inconsistent yields and protein measurements that were frequently higher than unprocessed BAL samples. Furthermore, ExoEasy preps contained many unique proteins and higher lipoproteins compared to SEC and UC. This may indicate false signal as a result of the EV isolation process making analysis and downstream applications unreliable without an additional buffer exchange step. Most of the protein in BAL was found in the non-EV fractions. In contrast, virtually all the nucleic acids, both RNA and dsDNA, found in BAL were in the protected environment of EVs.

**Conclusion:** SEC fractions containing EVs, when concentrated using 30 kDa centrifugal filters, yielded the lowest contaminating free protein and the highest nucleic acid content. This, coupled with good yield and preservation of EV structure and function for downstream use, makes it the ideal EV isolation protocol from BAL.

## INTRODUCTION

Extracellular vesicle (EV) research has been a rapidly growing field over the past two decades. Through this period, the International Society for Extracellular Vesicles was established and has since regularly produced guidelines for how to isolate and analyze EVs, titled “Minimal Information for Studies of Extracellular Vesicles” (MISEV)^[1]^. This guide summarizes EV characteristics and isolation from various sample types, including bacteria, blood, urine, cerebral spinal fluid, saliva, synovial fluid, milk, and solid tissue. Less common fluids, such as bronchoalveolar lavage (BAL), have been shown to contain EVs of varying clinical significance^[2]^, but there is no specific literature exists on how to optimize EV isolation from this unique fluid.

Direct analysis of BAL is important when trying to assess pulmonary immune and inflammatory responses. Numerous studies have highlighted that blood immune and inflammatory measurements are poor surrogates for what is occurring in the alveolar milieu^[3]^. However, analyzing BAL fluid presents several challenges, including the fact it requires an invasive procedure. BAL is a technique performed during fiberoptic bronchoscopy to isolate fluid and cellular contents of the alveolar space for microbial and cellular analysis. It provides both clinicians and scientists the opportunity to assess the inflammatory milieu of the alveolar space. When measuring pulmonary responses, one is technically evaluating the epithelial lining fluid (ELF) coating the alveolar surface. To obtain ELF, BAL is performed, which entails injecting normal saline into the lung followed by gentle aspiration to recover fluid^[4]^. This results in dilution of the ELF, typically by 33-100 times when dilution is corrected for using a urea dilution assay^[5,6]^. Despite this dilution, a large amount of protein remains present in BAL, especially albumin and immunoglobulins^[7,8]^, making separation of EVs from free protein and protein aggregates critical.

Substantial literature exists on EV isolation techniques from other biological samples, including a review article that describes different methods of EV isolation from BAL^[9]^. There has been no published attempt at optimization of the method for isolating EVs from BAL to maximize EV recovery and minimize contaminants that may influence or confound downstream assays.

Given the inherent dilution and variable nature of clinical samples, isolating EVs consistently and quantitatively from BAL can be challenging. In this work, we compared three published methods of EV isolation [ultracentrifugation (UC), size exclusion chromatography, and exoEasy (EE)] to determine the optimal protocol yielding consistent, quantifiable, and concentrated EVs from BAL with minimal free protein or cell debris contamination. We chose to study UC because many investigators consider this the gold standard for EV isolation^[10]^. EE was chosen as a representative of affinity column techniques. Size exclusion chromatography (SEC) was chosen because we hypothesized this isolation method would have the least effect on EV ultrastructure and have less contaminated contaminated by free protein aggregates.

## METHODS

### Specimen source and preparation

Two sources of frozen BAL were used for this study. The major source of BALs were from the Indiana University CLIA-approved Clinical BAL lab (*n* = 37), which performs cellular analysis for physicians to assist in diagnostic testing for various interstitial and alveolar lung diseases. The lab has Institutional Review Board (IRB) approval to utilize de-identified residual material for research purposes (IRB 1011003397). We intentionally chose to examine BALs with different inflammatory milieus (lymphocytic predominant, neutrophilic predominant, normal BAL differential) to improve the generalizability of the results. The second source of BAL samples came from prior National Institutes of Health-supported studies on healthy HIV-infected subjects on antiretroviral therapy and normal volunteers at Indiana University (*n* = 4). A summary of the BAL characteristics from both sources is shown in Supplementary Table 1. Importantly, while this represents a variety of clinical and research samples with differences in the inflammatory milieu between individual BALs, all subjects had BAL EV isolation using all three methods under study and thus served as their own controls. BAL samples are obtained and transported on ice to the BAL lab for processing and analysis^[11]^. Briefly, after running the sample through a 100 μm filter to remove large debris and mucus, BAL is centrifuged at 500 *g* for 10 min to obtain a cell pellet and acellular BAL supernatant. A cell differential is performed on a cytospin of the cell pellet for the referring physician. The acellular BAL fluid is stored at -80 °C and served as the material for EV isolation in this study. For experiments exploring dose response to isolation methods, 4, 2, and 1 mL of acellular BAL were used. For all other experiments, 1-2 mL of acellular BAL was used. Prior to EV isolation, stored acellular BAL samples were thawed and further clarified comparing two methods: filtration through a 0.65 um syringe filter or centrifugation at 10,000 *g* for 40 min. Finally, BAL needs to be reduced to 0.5 mL prior to loading on EE or SEC columns. This was performed using a 30 kDa Amicon filter.

**Table 1:** Top 25 proteins in extracellular vesicles obtained by size exclusion chromatography, UC, and EE

**Protein**	**SEC**	**UC**	**EE**
Albumin	X	X	X
Alpha-1 antitrypsin	X	X	X
Complement C3	X	X	X
Ubiquitin 60S ribosomal protein	X	X	X
Pulmonary surfactant-associated protein A2	X	X	X
Actin, cytoplasmic 1	X	X	X
Immunoglobulin heavy constant 1	X	X	X
Deleted in malignant brain tumors 1 protein	X	X	X
Immunoglobulin heavy constant gamma 3	X	X	X
Immunoglobulin kappa variable 3-20	X	X	X
Polymeric immunoglobulin receptor	X	X	X
Immunoglobulin heavy constant gamma 1	X	X	X
Serotransferrin	X	X	X
Pulmonary surfactant protein D	X	X	X
Complement C4-B	X	X	X
Beta-2 glycoprotein 1	X	X	
Immunoglobulin J chain	X	X	
Immunoglobulin lambda constant 3	X	X	
Immunoglobulin kappa constant	X	X	
Immunoglobulin heavy constant gamma 2	X	X	
Alpha-2 macroglobulin	X	X	
Galectin-3 binding protein	X	X	
Annexin A2	X		
Growth factor receptor bound protein 2	X		
Haptoglobin	X		
Histidine rich glycoprotein		X	
Pulmonary surfactant-associated protein B		X	
BPI fold containing family B member 1		X	X
Apolipoprotein A-I			X
Uteroglobin			X
CD 44 antigen			X
Calmodulin			X
Transthyretin			X
Plasma protease C1 inhibitor			X
Protein AMBP			X
Kinogen-1			X
Inter-alpha-trypsin inhibitor heavy chain A2			X

“X” means this protein was found in proteomics analysis using this EV isolation method. EVs: Extracellular vesicles; SEC: size exclusion chromatography; UC: ultracentrifugation; EE: exoEasy; CD: cluster of differentiation; AMBP: alpha-1-microglobulin/bikunin precursor; BPI: bactericidal/permeability-increasing protein.

### UC protocol

Filtered acellular BAL samples were centrifuged at 43,000 rpm on Beckman SW55Ti rotor (minimum~125,572 *g* and maximum 224,089 *g* in swinging bucket rotor), supernatants were discarded and Gibco Dulbecco’s phosphate buffered saline(DPBS) was added to wash pellets followed by an additional 43,000 rpm centrifugation. Supernatants were again discarded with EV containing pellet resuspended in appropriate amount of DPBS.

### EE protocol

Filtered and concentrated acellular BAL was processed using Qiagen EE Maxi kit according to manufacturer’s provided protocol: Equivalent volume of provided buffer XBP was added to sample and gently mixed by inverting tube 5 times and allowing the sample/XBP solution to come to room temperature. This solution was then added to the provided EE spin column and centrifuged at 500 × *g* for 1 min at room temperature with flow through discarded. The spin column containing bound EVs was then washed with 10 mL provided buffer XWP and centrifuged at 5,000 × *g* for 5 min at room temperature two times with flow through discarded after each centrifugation. The spin column was then transferred to new provided collection tube with 250 µL provided buffer XE (elution buffer) added and incubated for 1 min at room temperature prior to centrifugation at 500 × *g* for 5 min at room temperature. The eluate was then reapplied to the column and incubated at room temperature for 1 min and then centrifuged at 5,000 × *g* for 5 min at room temperature to maximize column yield as indicated in provided kit instructions.

### Size exclusion chromatography

Filtered and concentrated acellular BAL was loaded into Izon 70 nm qEV Original gen 2 SEC columns. Eluted fractions were collected utilizing Izon Automated Fraction Collector with settings of Buffer Volume 2.7 mL and Fraction Volume 0.4 mL. Up to 30 fractions were collected. Fractions 1-6 contain EVs and were pooled for further analysis. Fractions 7 and higher were considered “non-EV containing” fractions.

### Size exclusion chromatography fraction concentration

Eluted EV fractions 1-6 were too dilute and needed to be concentrated for further studies. To determine the optimal concentration method, equal volumes of pooled fraction 1-6 were concentrated by multiple methods: uc at 43,000 rpm on a Beckman SW55Ti rotor (as described above); exoEasy per the manufacturer’s protocol; and Amicon centrifugal filters with molecular weight cutoffs of 100, 50, 30, and 10 kDa.

### Protein concentration assay

Samples were measured for total protein using ThermoFisher Pierce bicinchoninic acid (BCA) Assay on microplate protocol the manufacturer’s protocol (Cat No. 23225).

### Nucleic acid analysis

Samples were treated with 0.1% Triton X-100 and incubated at room temperature for 30 min prior to analysis. RNA was quantified by ultraviolet (UV) spectrophotometry utilizing Thermo Scientific NanoDrop One Microvolume UV-Vis Spectrophotometer (ND-ONE-W), utilizing 0.1% Triton X-100 as a blank as a blank to account for possible background from lysis buffer. EV nucleic acid content for RNA and dsDNA was also measured using an Invitrogen^TM^ Quant-iT^TM^ RNA Assay Kit (Cat No. Q33140) and Invitrogen^TM^ Quant-iT^TM^ PicoGreen^TM^ dsDNA Assay Kits (Cat No. P7589).

### Optimization of EV content quantification

Since EVs have both membrane-bound and intravesicular contents, we performed studies to determien whether various solubilization methods imrpoved. Acellular filtered BAL samples were collected via size exclusion chromatography as described above and then concentrated by 30 kDa Amicon centrifugal filters. Samples were then lysed using either Triton-x 100 0.1% (Cat No. A16046.AE), Cell Lysis Buffer from Cell Signaling Technology (Cat No. 9803S), or RIPA Buffer from Thermo Fisher Scientific (Cat No. 89900) and assessed for protein via BCA Assay and nucleic acid content by UV spectrophotometry.

### Western blots

Samples were prepared with Invitrogen Bolt 4 × Sample Loading Buffer (Cat No. B0007) and 10 × Bolt Sample Reducing Agent (Cat No. B0009) according to manufacturer’s protocol. Samples were then loaded onto 1.0 mm neutral polyacrylamide gel electrophoresis (NuPAGE) Bis-Tris Mini Protein Gels 4%-12% (Cat No. NP0321-4BOX) and run according to manufacturer’s protocol. Protein from the gel was then transferred to Bio-Rad Immun-Blot polyvinylidene difluoride (PVDF) membrane (Cat No. 1620177) using Bio-Rad Trans-Blot Turbo Transfer System. Western Blotting then was completed according to Bio-Rad Western Blotting Protocol utilizing Bio-Rad 10 × tris-buffered saline (TBS) (Cat No. 1701706435), Tween 20 (Sigma Aldrich Cat No. P1379-100 mL), and Blotto, non-fat dry milk (ChemCruz Cat No. sc-2324 250 g) to create the 5% Milk-TBST utilized in Blocking steps. EV transmembrane primary antibodies were obtained from Cell Signaling: Syntenin-1/melenoma differentiation associated gene-9 (MDA9) (E2I9L) Rabbit mAb (Cat No. 27964S, lot 1), cluster of differentiation (CD81) (D3N2D) Rabbit mAb (Cat No. 56039S, Lot 2), CD63 (E1W3T) Rabbit mAb (Cat No. 52090S, Lot 1), and CD9 (D3H4P) Rabbit mAb (Cat No. 13403S, lot 5). The EV intracellular protein tumor susceptibility gene (TSG101) rabbit mAb was obtained from Invitrogen (Cat No. PA5-31260). Invitrogen was the source of goat anti-rabbit secondary Ab (Cat No. 31460, Lot ZA387781) and Super Signal West Femto Maximum Sensitivity Substrate (34096, lot ZG394565). Images were obtained and processed using Bio-Rad ChemiDoc Touch Imaging System.

### Nanosight particle tracking

Nanosight LM-10 by Malvern Panalytical along with nanoparticle tracking analysis (NTA) 3.3 software was utilized to quantify particles according to manufacturer’s instructions^[12,13]^.

### Transmission electron microscopy

Transmission electron microscopy was performed as previously described^[14]^. Briefly, a few microliters of large extracellular vesicle (LEVs) and small extracellular vesicle (SEVs) were suspended in phosphate buffered saline (PBS), adsorbed on the carbon-coated copper grid by floatation for 20 min, and washed with deionized water six times followed by 1% uranyl acetate staining to enhance the contrast. For the IZON SEC samples, the sample was used directly without any dilution and floatation was performed for 2 min followed by washing and staining. The images were acquired using transmission electron microscopy at 100 kV emission (JEM-1400, Jeol, USA). Images were then analyzed for particle size and morphology using Image J software with scales set appropriately. The diameter of each particle was manually measured. Spherical cupped shaped particles were considered EVs. Smaller non-EV particles, appearing as dark circles without central clearing particles were also measured and all particles measured < 30 nm were considered non-EV contaminants. Between 3 and 5 random fields of viewed at 1,000 × were analyzed for each sample to generate total particle counts, and between 6 and 12,3000 × and 10,000 × images were analyzed for discrete particle size measurement and confirmation of morphology for each 1,000 × field analyzed.

### Proteomics

Sample preparation, mass spectrometry analysis, bioinformatics, and data evaluation for quantitative proteomics experiments were performed in collaboration with the Indiana University School of Medicine Center for Proteome Analysis similarly to several previously published protocols^[15,16]^. Briefly, EV preparations obtained by SEC, UC, and EE from BAL from two subjects were lysed in 8 M Urea and digested with Trypsin Gold/LysC (Promega) and mass spectrometry was performed utilizing an EASY-nLC 1200 high-performance liquid chromatography (HPLC) system coupled to Exploris 480^TM^ mass spectrometer with FAIMSpro interface (Thermo Fisher Scientific). Data were analyzed using Proteome Discoverer 2.5.0.400 (Thermo Fisher Scientific). A Homo sapiens reviewed proteome database (UniProtKB; 20292 sequences downloaded 05132022), plus common laboratory contaminants (73 sequences including streptavidin) was searched using SEQUEST HT. Percolator false discovery rate (FDR) filtration of 1% was applied to both the peptide-spectrum match and protein levels. In the consensus workflow, the precursor ion intensity of unique and razor peptides was used for protein quantification with summed abundances. Only proteins with greater than 2 unique peptides identified were used for various downstream comparisons and pathway analyses. Protein-protein association networks and functional enrichment analyses were performed using the STRING database^[17]^.

## RESULTS

### Sample preparation prior to EV isolation

The two most common methods of removing large particles from EVs prior to UC, SEC, or EE are using sterile syringe filters (0.65 micron) or a 40 min high speed centrifugation at 10,000 *g*. We directly compared these two methods, and found that EV particle yields and tetraspanin concentrations were identical [Supplementary Figure 1]. We chose use of the 0.65 um filter for three reasons. First, it is significantly easier and requires less time. Second, we were attempting to include microvesicles and larger exosome populations which might be lost during centrifugation in our preparation. Thirdly, use of a filter is less likely to cause structural damage to EVs compared to high speed centrifugation^[18]^.

**Figure 1:**
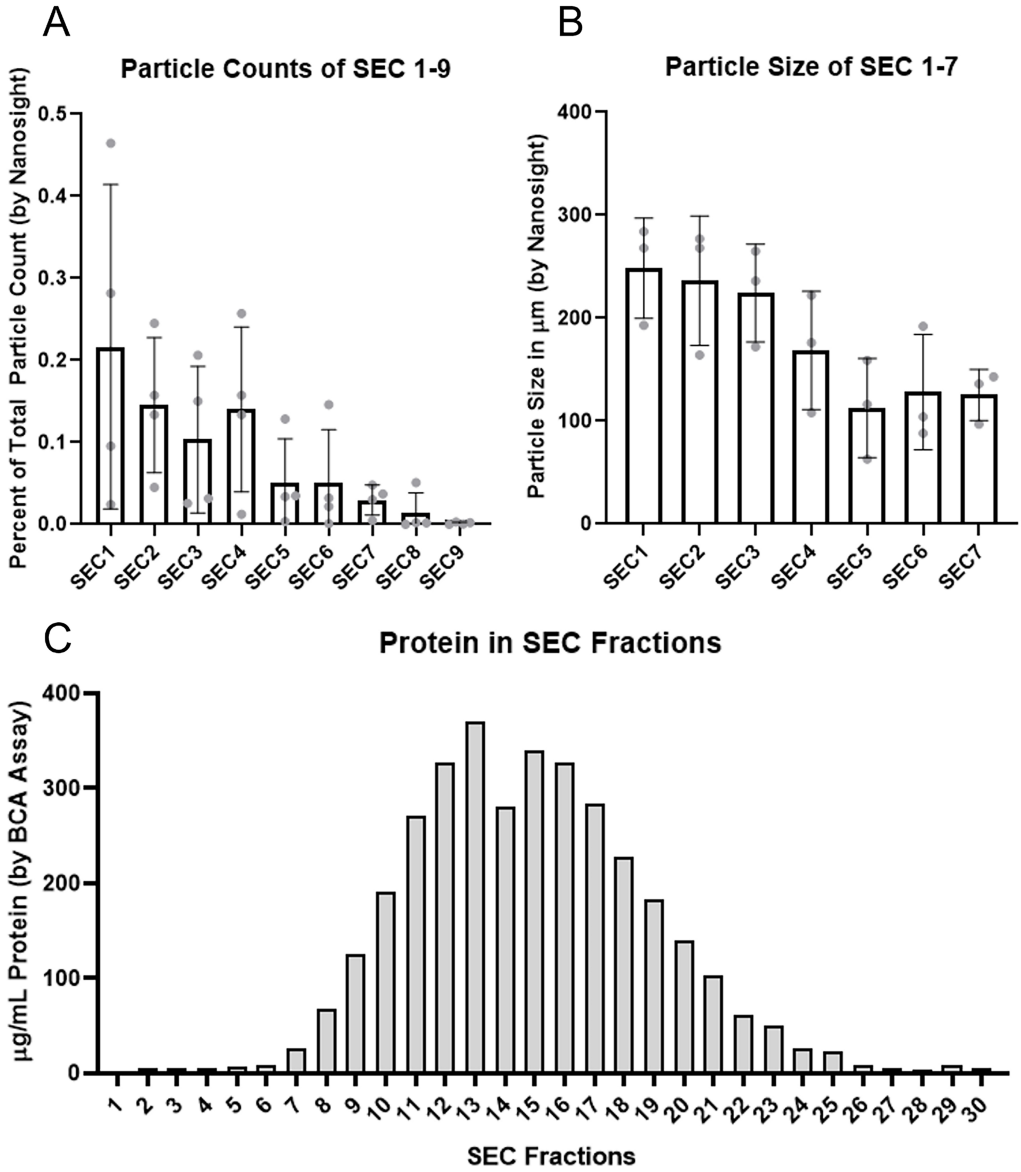
Size exclusion chromatography fraction particle counts, size, and protein content. (A) Particle counts represented as a fraction of total sample particle count as determined by Nanosight LM-10 of SEC Fractions 1-9 demonstrating decrease in particle counts in later fractions until being below the limit of detection by Nanosight in SEC fraction 9, *n* = 4 subjects, error bars represent standard deviation; (B) Particle size of particles contained within SEC fractions 1-7 demonstrating decrease in particle size with increase in SEC fractions, *n* = 3 subjects, error bars represent standard deviation; (C) Total protein count in SEC fractions 1-30 demonstrating minimal protein in SEC fractions 1-6 with increase in protein concentration in fractions 7-25 with a peak in fraction 13, *n* = 1 subject. SEC: Size exclusion chromatography; LM-10: Nanosight LM-10 particle analysis system; SD: standard deviation.

### Optimization of size exclusion chromatography

Initial investigations focused on determining which fractions to collect after discarding the buffer volume. Fractions 1-7 yielded particles/frame that were able to be accurately quantified by Nanosight with 88.7% of measured particle contained within fractions 1-6 [Figure 1A]. In addition, there was a predicted gradual reduction in average particle size with each subsequent fraction [Figure 1B]. Assessment of total protein concentrations revealed fractions 1-6 had minimal protein relative to the other fractions and measurable protein started to increase at fraction 7 with a peak at fraction 13 [Figure 1C].

**Figure 2:**
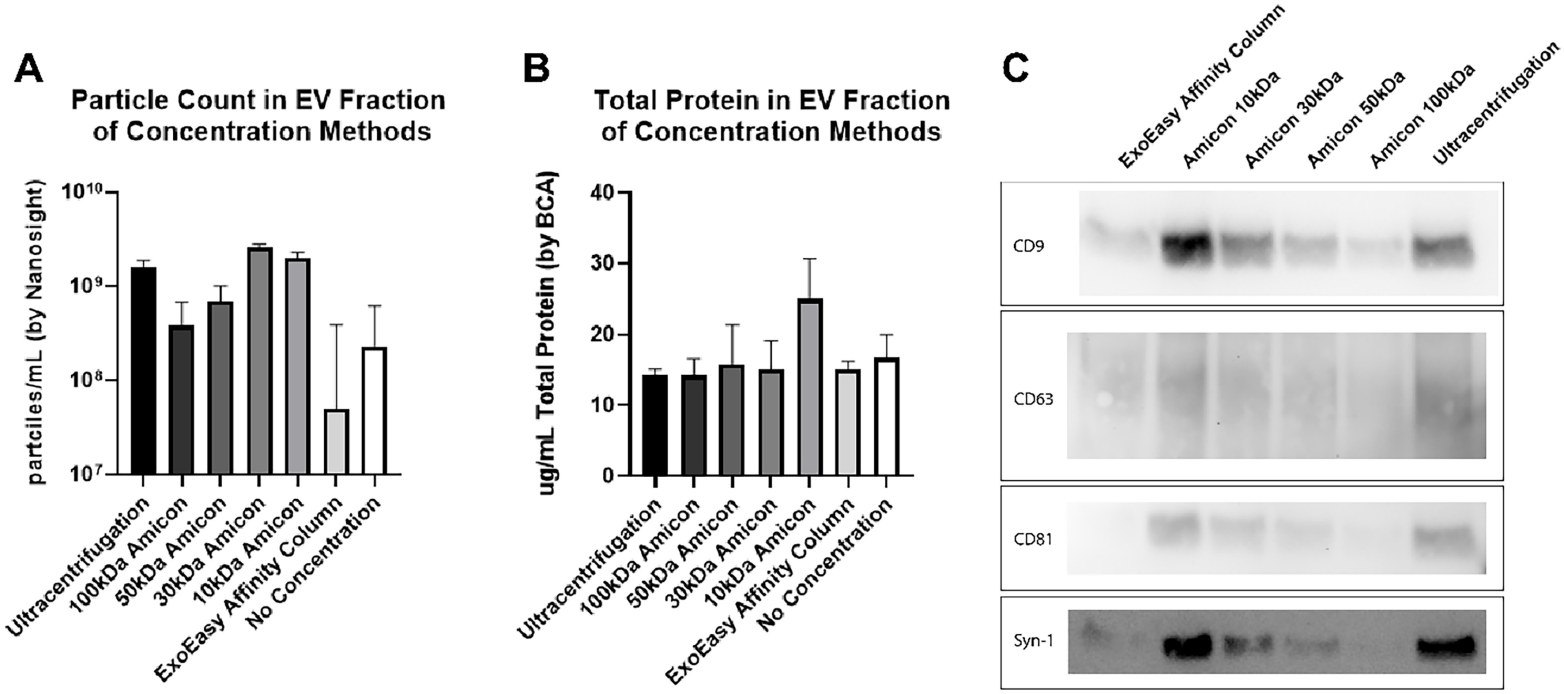
Characterization of BAL EVs isolated by SEC and concentrated by various methods. Particle count as determined by Nanosight (A) and total protein by BCA (B) in each concentration method. Concentrating SEC fractions 1-6 using a 30 kDa Amicon centrifuge filter yielded equally high EV particles compared to concentration using 10 kDa centrifuge filter with lower free protein contamination. *n* = 1 subject run three times to demonstrate technique reproducibility. Error bars represent SEM as determined by NTA 3.3 software from Malvern Paranalytical; (C) Western Blot of Tetraspannins CD9, CD63, CD81, and Syn-1 of EVs isolated by SEC and concentrated by either Amicon centrifuge filters, EE kit, or ultracentrifugation. BAL: Bronchoalveolar lavage; EVs: extracellular vesicles; SEC: size exclusion chromatography; BCA: bicinchoninic acid; kDa: kilodalton; SEM: standard error of the mean; NTA: nanoparticle tracking analysis.

SEC introduces large buffer volumes to the samples during fractionation, which dilutes the sample significantly. To be useful in downstream applications, the fractions collected require a concentration step. Concentration of pooled SEC fractions 1-6 was compared using Amicon filters (10, 30, 50, and 100 kDa), UC, and EE kits. The 30 kDa Amicon filter had the best yield when concentrating samples after SEC using Nanosight particle tracking [Figure 2A]. The superior yield compared to larger kDa filters may reflect smaller EVs passing through or getting stuck on the surface of the filter. Note that concentrating SEC fractions 1-6 using a 30 kDa Amicon centrifuge filter yielded equally high EV particles compared to concentration using a 10 kDa centrifuge filter but with significantly lower free protein contamination [Figure 2B]. EV enrichment was confirmed by western blotting for Syntenin-1 and the tetraspanins CD9, CD63, and CD81 [Figure 2C]. The darker bands observed with the 10 kDa filter and ultracentrifugation further highlight free protein contamination, as particle counts were similar across methods.

### Direct comparison of the three EV isolation methods

**Figure 3:**
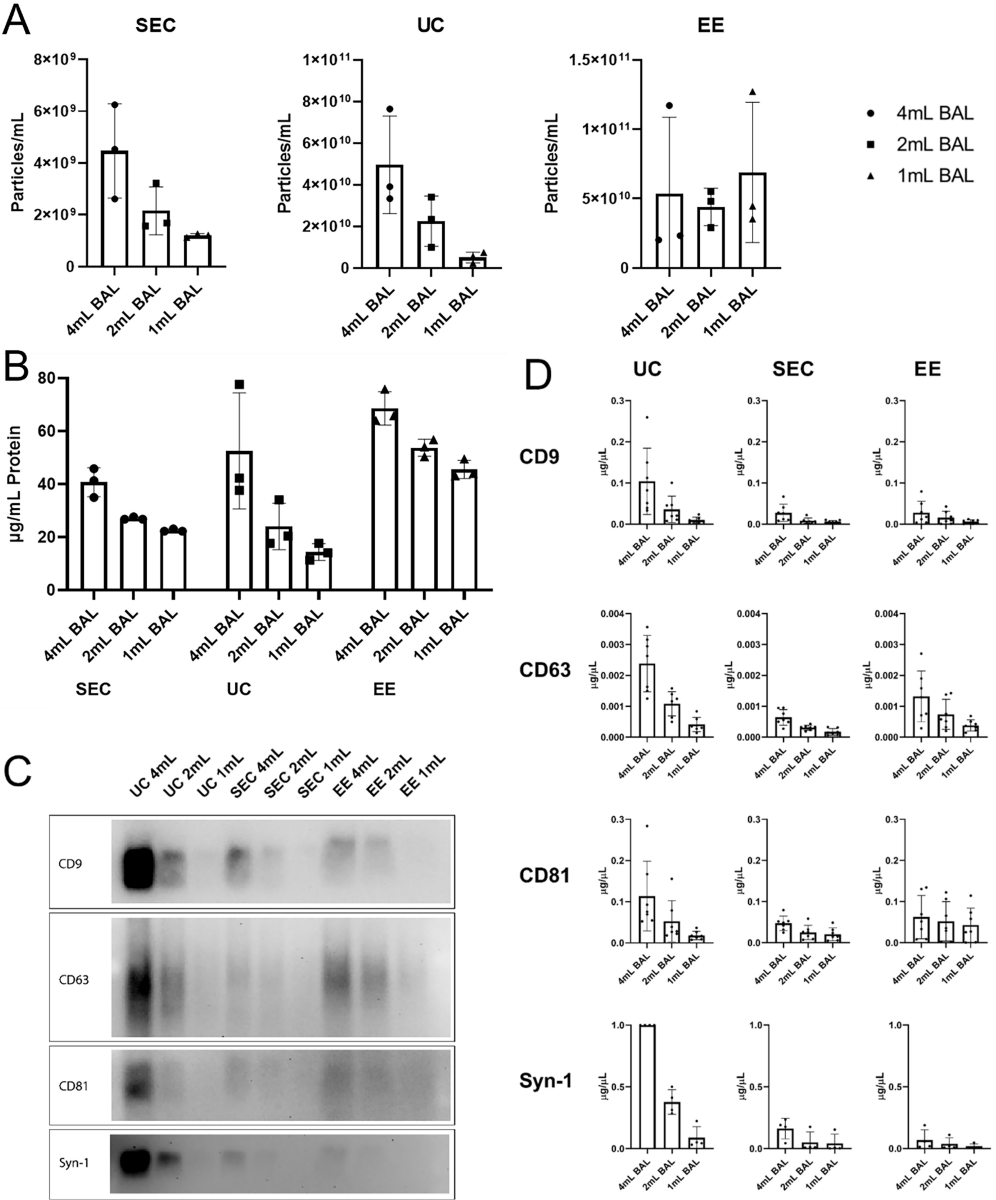
Quantification of EVs isolated by ultracentrifugation, size exclusion chromatography, or EE affinity column. EVs were isolated from 4, 2, and 1 mL of acellular BAL by SEC, UC, and EE. Preparations were then analyzed for (A) particle yield by Nanosight (*n* = 3 subjects); (B) protein concentration (*n* = 3 subjects); (C) assessment of EV transmembrane proteins tetraspanin and syntenin-1 by western blotting; (D) Quantitation of western blot bands (*n* = 3 subjects); (E) Further confirmation of EV isolation was performed by detection of the intracellular EV protein TSG101. EVs: Extracellular vesicles; BAL: bronchoalveolar lavage; SEC: size exclusion chromatography; UC: ultracentrifugation; EE: exoEasy; CD: cluster of differentiation; Syn-1: syntenin-1; TSG101: tumor susceptibility gene 101.

For these experiments, equal amounts of BAL (4, 2, and 1 mL) were processed using each of the isolation methods and particle counts and protein concentrations were measured [Figure 3A]. Using Nanosight particle tracking, EE and UC yielded higher particle counts compared to SEC. However, there was not an appropriate gradient based on the amount of BAL processed using EE despite being loaded with material far below the columns upper threshold as indicated in the published protocol. In contrast, both SEC and UC gave nice dose responses to different amounts of BAL being processed. UC yielded on average one log higher particle count by Nanosight compared to SEC. Analysis of protein concentrations in the EV preparations also showed the highest amounts when EE columns were used [Figure 3B], suggesting contamination of EV preps by proteins from the column or protein aggregates. The SEC method yielded the least total protein overall and changed proportionally to the amount of BAL processed. Furthermore, when compared to UC, SEC demonstrated far less sample-to-sample variability as evidenced by the very narrow error bars.

**Figure 4:**
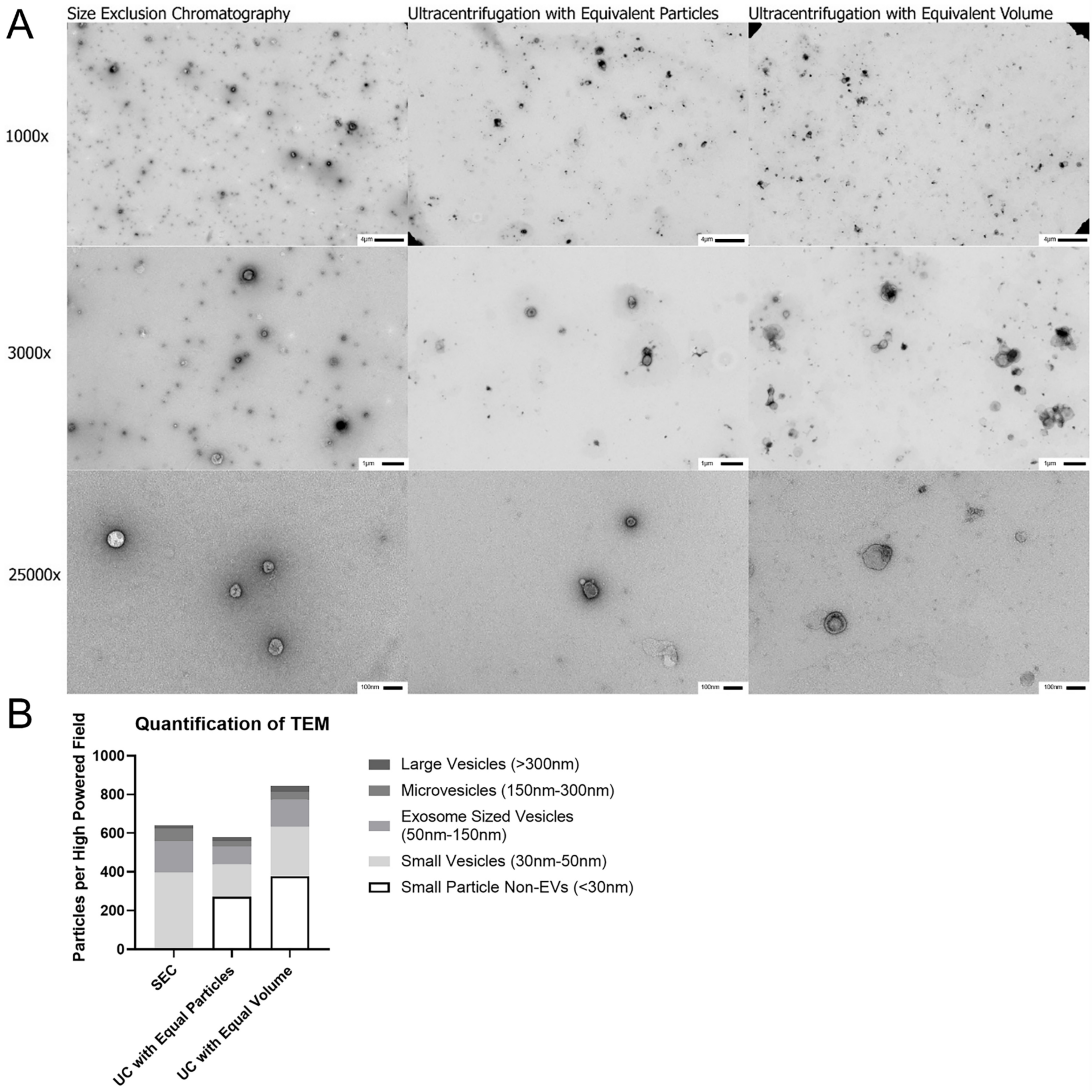
Transmission electron microscopy of EVs isolated from BAL by SEC and UC. (A) EVs were isolated by SEC and UC and preparations imaged using both equivalent particle counts by Nanosight LM-10 and equal volume from original acellular BAL to visualize EV yield, 1,000 ×, 3,000 ×, and 25,000 × magnifications are shown. All samples imaged demonstrate typical cupping that is characteristic of EVs, but the SEC sample shows higher density of identifiable EVs per High Powered Field; (B) 1,000 × images were used to quantify number of particles with 3,000 × and 25,000 × magnifications utilized to clarify size and nature of particles. Non-EV particles were not included in the counts as determined by lack of central cupping on higher magnification images. SEC demonstrated significantly higher quantities of small particles EVs and the Exosome population compared to both UC preparations. *n* = 3-5 HPF for 1,000 × and 5-9 HPF for 3,000 × and 25,000 × magnifications. EVs: Extracellular vesicles; BAL: bronchoalveolar lavage; SEC: size exclusion chromatography; UC: ultracentrifugation; TEM: transmission electron microscopy; HPF: high power field.

Western blotting for EV transmembrane proteins Syntenin-1 and tetraspanins was performed to confirm the presence of EVs in the preparations. Interestingly, despite having the most EVs by particle tracking, EE preparations contained only small amounts of detectable Syntenin-1 and the tetraspanins CD9, CD63, and CD81 [Figure 3C and D]. UC yielded the largest amount of these proteins on western blotting [Figure 3C and D]. Further confirmation of EV isolation was demonstrated by detection of the intracellular EV protein TSG101 [Figure 3E]. Given concerns about protein contamination with UC, transmission electron microscopy was performed on UC and SEC EV preparations [Figure 4A]. Quantitative analysis demonstrated that SEC yielded more true EVs compared to UC regardless of whether the starting material was corrected for starting volume or the number of EVs as determined by Nanosight [Figure 4B]. These experiments demonstrated that there were not only notably more EVs seen in SEC samples, but there was also less non-EV material present.

### Analysis of EV and non-EV content

**Figure 5:**
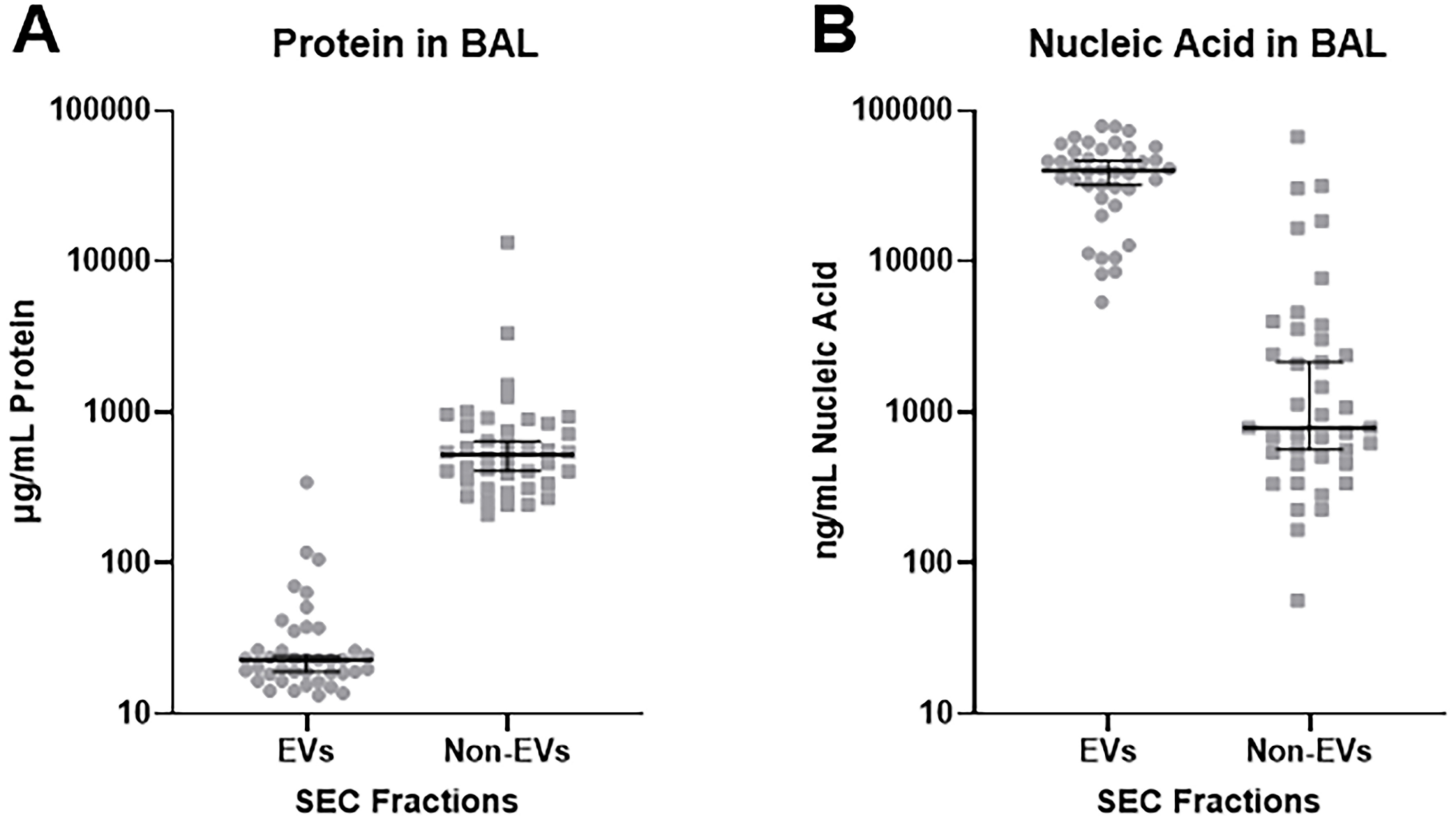
Total protein and nucleic acid in EV and non-EV fractions collected from SEC as determined by BCA assay and NanoDrop. (A) EVs isolated from acellular BAL contain significantly less protein than the non-EV fraction (*P* = 0.0009); (B) EVs isolated from acellular BAL contain significantly more nucleic acid material than the non-EV fraction (*P* < 0.0001). *n* = 40 subjects. Analysis performed using unpaired two-tailed t-test assuming equal variance. EVs: Extracellular vesicles; SEC: size exclusion chromatography; BAL: bronchoalveolar lavage; BCA: bicinchoninic acid.

Next, we wished to further characterize the contents of EV and non-EV fractions obtained by SEC. SEC fractions 1-6 were concentrated using a 30 kDa Amicon filter. Fractions 7 and above were also pooled and concentrated. As expected from Figure 1C, the majority of protein detected in BAL by BCA assay (95.9%) is in the non-EV fraction [Figure 5A]. All three EV isolation methods similarly removed the majority of free protein from bronchoalveolar lavage [Supplementary Figure 2]. In contrast, using NanoDrop spectrophotometry, we found that 89.2% of nucleic acids are contained within EV fraction [Figure 5B]. We also tested whether treating EVs with various detergents to lyse membranes would increase protein content. Supplementary Figure 3 demonstrates that treating EVs with various membrane lysis agents increased expression of syntenin-1 and all the tetraspanins. This figure also demonstrates that EVs were only contained in SEC fractions 1-6, as all the remaining pooled and concentrated SEC fractions (7-30) demonstrated no syntenin-1 and tetraspanin signaling on western blotting.

### Comparison of protein and nucleic acid content in EV preparations using all three isolation methods

**Figure 6:**
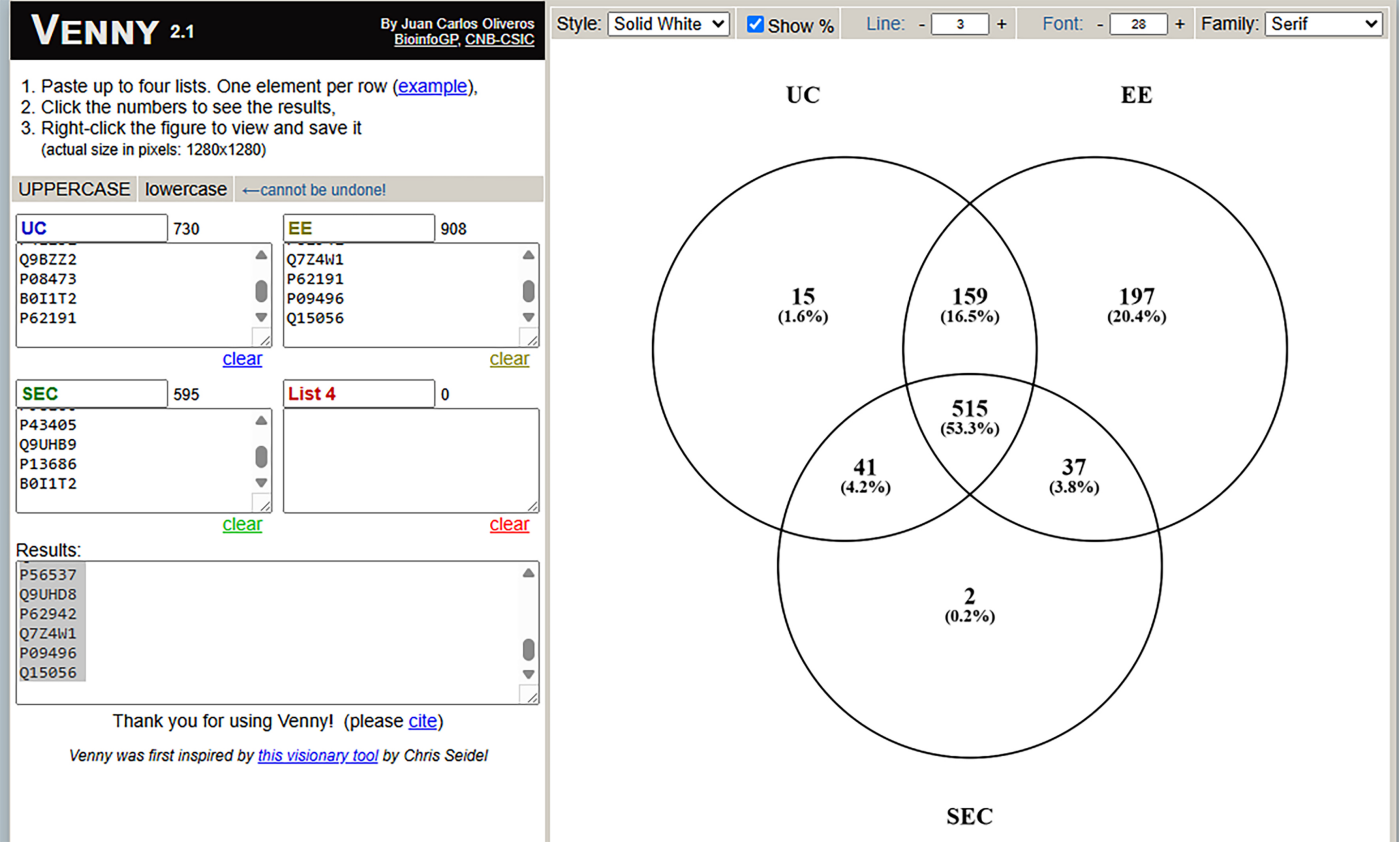
Venn diagram demonstrating the number of unique and overlapping proteins found in different extracellular vesicle preparations. EV preparations obtained from BAL by SEC, UC and EE from two subjects underwent bottom up label free mass spectrometry and proteins identified with high confidence and with greater than 2 unique peptides were compared across the preparations mass spectrometry. EVs: Extracellular vesicles; BAL: bronchoalveolar lavage; SEC: size exclusion chromatography; UC: ultracentrifugation; EE: exoEasy.

Finally, we determined if different EV isolation methods resulted in different types of proteins and nucleic acids found in the preparations. First, we used mass spectrometry based proteomic approaches to compare proteins found in EV preparations from all three methods. The Venn diagram in Figure 6 shows that 595 proteins were identified in SEC EV preps, 730 proteins were identified in UC EV preps, and 908 proteins were identified in EE EV preps. A total of 515 proteins were found in all three preparations. Note very few proteins were uniquely found in the SEC EV prep, while many proteins were identified only in the EE prep. Table 1 shows the top 25 most abundant proteins in each preparation based on summed protein ion abundance. There is good agreement between the SEC and UC EV preparations. The EE preparation had numerous unique proteins identified which were not seen in the other two EV preps.

**Figure 7:**
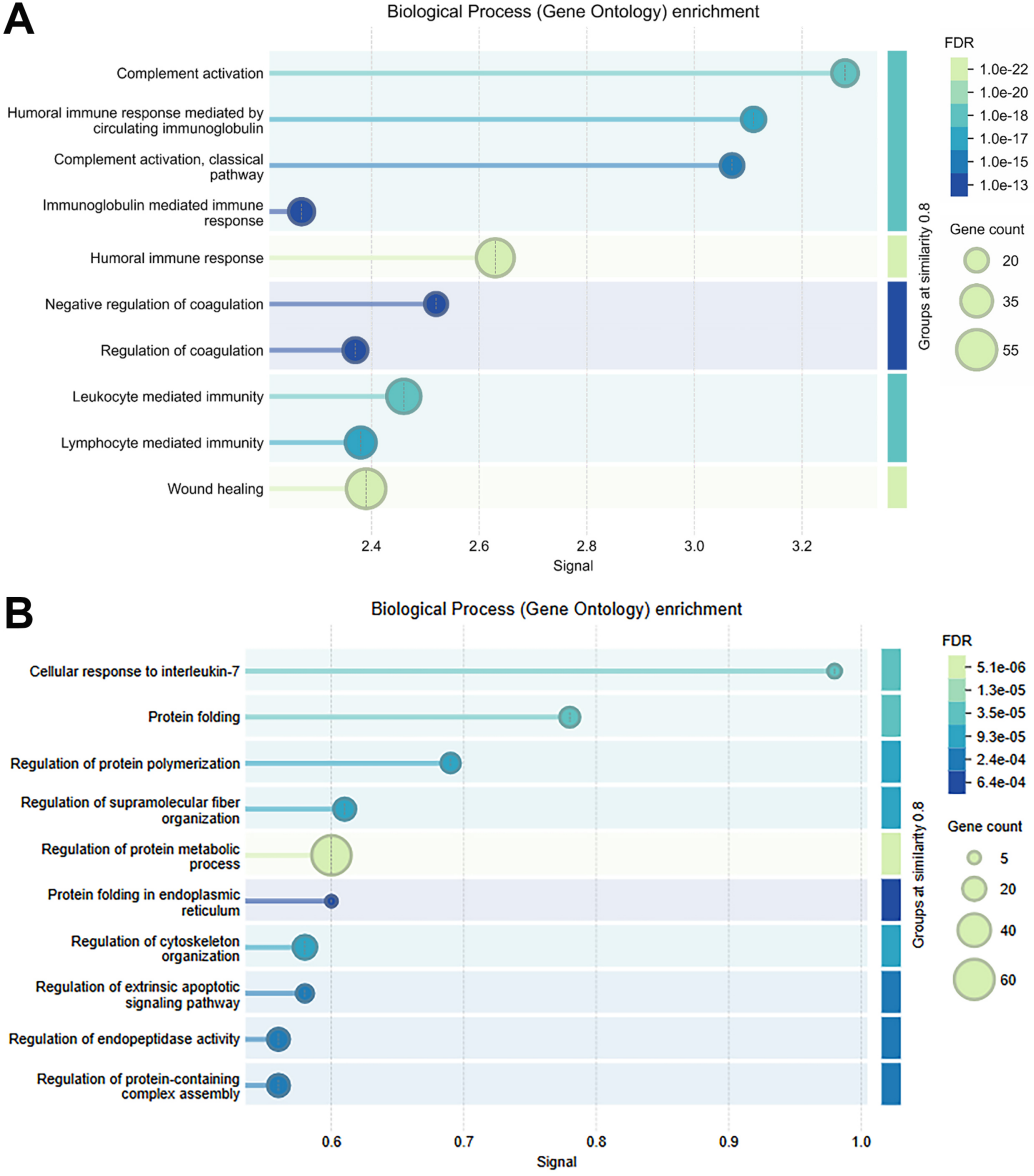
Summary of gene ontology enrichment analyses. Analysis was performed using the STRING 12.0 database on (A) the 515 proteins found with high confidence in all EV preparations and on (B) the 197 proteins uniquely found in the ExoEasy EV preparation. EV: Extracellular vesicle; STRING: Search Tool for the Retrieval of Interacting Genes/Proteins.

We also performed pathway enrichment analysis of the 515 proteins found in all three EV preparations using the STRING database 12.0 [Figure 7A]. These proteins were strongly slanted towards immunologic and inflammatory responses. This likely reflects that our cohort was made up of BALs from either HIV-infected subjects or subjects undergoing a clinical BAL to evaluate potential lung inflammation. Interestingly, pathway analysis of the 197 proteins found exclusively in the EE EV preparation [Figure 7B] were more aligned with intracellular processes like protein folding, supramolecular fiber integration, protein metabolic process, protein folding in endoplasmic reticulum (ER), regulation of cytoskeleton, and regulation of apoptotic signaling pathway. This suggests that intracellular debris and apoptotic bodies were being recovered in EE EV preparations.

It is well known that EV preparations may also contain lipoproteins^[19]^. We used proteomic data to validate the presence of apolipoproteins, which bind to lipoproteins and thus serve as a good surrogate marker for lipoproteins in our EV preparations^[20,21]^. Table 2 demonstrates that all three EV preparations had apolipoproteins, suggesting lipoprotein recovery. However, these proteins were significantly more abundant in EE EVs as compared to UC and size exclusion chromatography preps.

**Table 2:** Relative abundance of apolipoproteins found in EV preparations using UC, EE, and SEC

	**Abundance ratios**		
	**EE/SEC**	**EE/UC**	**SEC/UC**
**Apolipoprotein A-I**	14.207	15.718	1.056
**Apolipoprotein A-IV**	16.347	13.203	0.862
**Apolipoprotein B-100**	5.283	1.153	0.235
**Apolipoprotein E**	6.593	5.338	0.735
**Apolipoprotein A-II**	25.031	10.621	0.506
**Apolipoprotein CI**	3.891	9.230	2.796
**Apolipoprotein C-III**	14.490	26.475	13.247
**Apolipoprotein D**	32.653	2.439	0.051
**Apolipoprotein C2**	100.000^*^	100.000	
**Apolipoprotein B receptor**	100.000	30.895	0.010

^*^is a surrogate value for infinity. EVs: Extracellular vesicles; UC: ultracentrifugation; EE: exoEasy; SEC: size exclusion chromatography.

**Figure 8:**
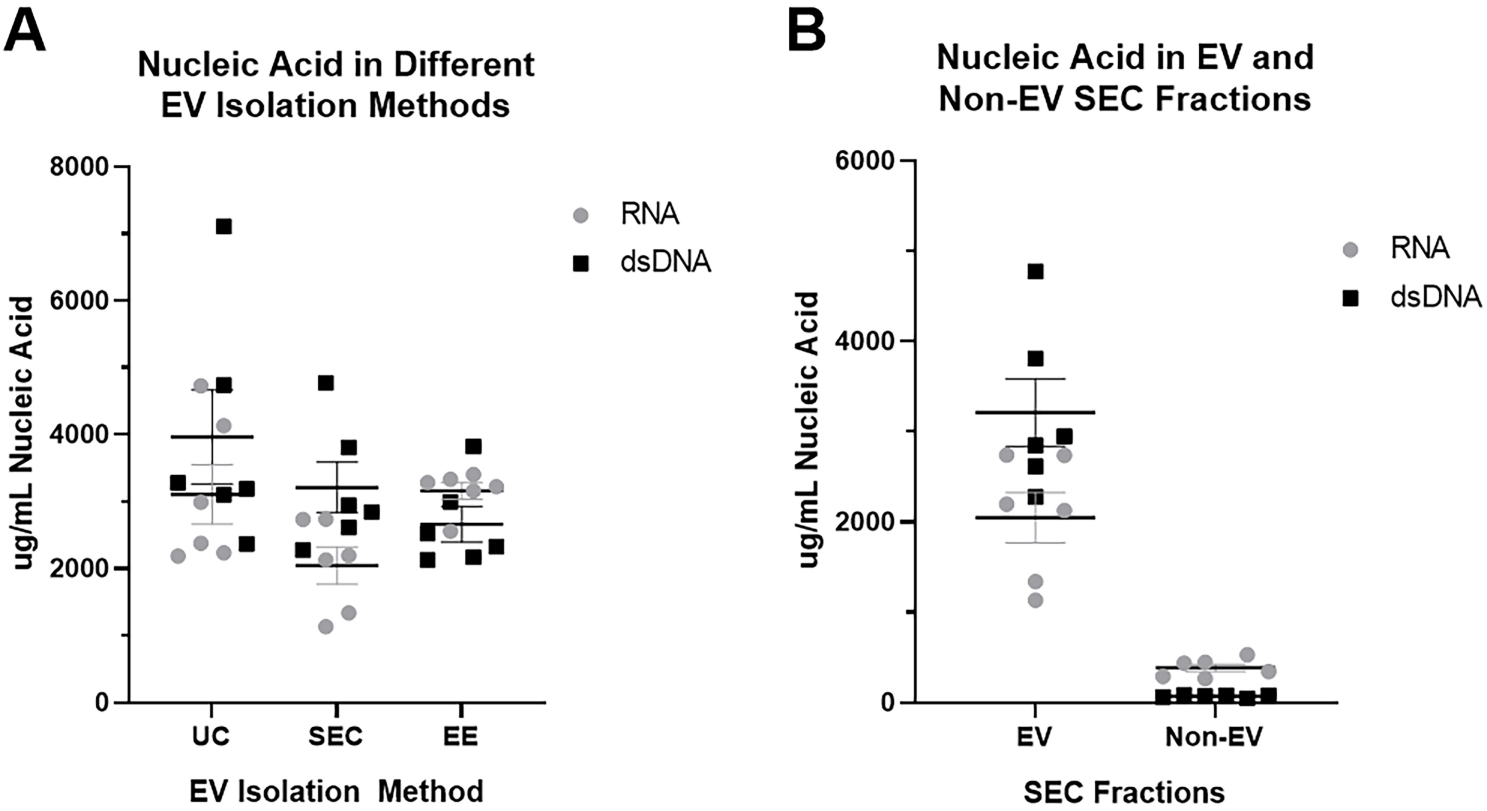
Measurement of RNA and dsDNA in nucleic acids isolated from extracellular vesicle preparations. EV nucleic acid content for RNA and dsDNA was measured using Invitrogen^TM^ Quant-iT^TM^ RNA Assay and Invitrogen^TM^ Quant-iT^TM^ PicoGreen^TM^ dsDNA Assay Kits. (A) Comparison of RNA and dsDNA in EV preparations using SEC, UC, and EE; (B) Comparison of RNA and dsDNA found in the EV and non-EV fractions obtained by size exclusion chromatography. *n* = 2 subjects run in triplicate. EV: Extracellular vesicle; RNA: ribonucleic acid; dsDNA: double-stranded deoxyribonucleic acid; SEC: size exclusion chromatography; UC: ultracentrifugation; EE: exoEasy.

Finally, we further characterized the nucleic acids in our EV preps using a kit to distinguish RNA and dsDNA. Extracellular vesicle nucleic acid content was similar for RNA and dsDNA using all three EV isolation methods [Figure 8A], demonstrating that all three methods (including SEC) were adequate to isolate EV nucleic acids. Figure 8B further demonstrates that most of the nucleic acid in BAL was found in EVs compared to the non-EV fraction.

## DISCUSSION

While there are many published methods to isolate EVs, each has its advantages and disadvantages^[22]^. UC is widely used, but has been demonstrated to have variable EV yields and has the potential to physically damage EVs^[18,23-25]^. Size Exclusion Chromatography^[26]^ is quick, has a high-yield, and EV integrity is maintained through the isolation process^[24]^. Size exclusion does have some disadvantages. Depending on fractions collected and column used there may be non-EV contaminants. Furthermore, the final preparation is significantly diluted. These disadvantages are mitigated with the use of a concentration step^[27]^. Precipitation has high EV yield, but preparations contain non-EV contaminants which may prevent downstream use due to polymer formation, limiting functionality of EVs^[28-31]^. Immunocapture can yield pure, but low yield EV populations and does not isolate all EVs^[32,33]^. Ultrafiltration is easy to perform and can be combined with other methods, but has variable yield and composition^[34,35]^. Field-flow fractionation can separate EVs into subset fractions, but can not be utilized as a standalone method^[22]^. Microfluidics can isolate EVs from small samples and isolate subpopulations, but is not suitable for preparative purposes, limiting downstream use and application^[36]^. Finally, membrane affinity binding can be used to isolate all EVs, but cannot discriminate between EVs and cell membrane fragments or apoptotic bodies^[22]^.

In this work, our aim was to determine the best way to isolate EVs from BAL, which has the additional confounding property of being an inherently dilute biological specimen^[6]^. Our goals were to identify a method that yields high EV quantities from dilute starting material, produce preparations with minimal free protein contamination, and maintain EV structural integrity for downstream applications. With these goals in mind, we chose to compare three distinct methods of EV isolation: membrane-based affinity chromatography (EE), standard UC, and size exclusion chromatography coupled with various concentration steps. Our results demonstrate that SEC, while not always yielding the highest quantities, provides final preparations that have the least free protein contamination and maintain the structural integrity of the EVs. This method also allows one to directly compare compounds of interest in EV and non-EV fractions. Analysis of protein and nucleic acid contents in EV and non-EV fractions are highly consistent and predictable. As expected, most protein in BAL was found in the non-EV fraction, consistent with the known abundance of free protein, especially albumin and immunoglobulins, in BAL fluid^[37,38]^. In contrast, our results add to that of other investigators^[39]^ demonstrating that the bulk of nucleic acids in BAL are found in EVs, which serves as a protected environment against RNAses and DNAses found in most biological specimens^[40]^.

Isolation of EVs from biologic specimens requires careful consideration of sample preparation before, during, and after EV isolation. Prior to isolating EVs, one must first remove large subcellular debris, organelles, and apoptotic bodies. We found that passing the specimen through a 0.65 μm syringe filter yielded higher EV loads compared to centrifugation at 10,000 *g* for 40 min prior to SEC column loading. During isolation of EVs from the SEC column, prior publications suggested that fractions 1-5 or 2-5 should be collected to maximize EV yield^[41,42]^. From our Nanosight data, fraction 1 had the second highest concentration of particles, and excluding this fraction would decrease yield significantly as well as possibly exclude the microvesicle population of vesicles. Our results verified that the majority of particles detected by Nanosight are contained within these fractions and to maximize yield, fractions 1-6 can be collected with minimal contaminating protein as determined by BCA Assay. Finally, after collecting the EV fractions, concentration of the large elution volume is necessary. Our results indicate that use of a 30 kDa Amicon filter to concentrate EV fractions had the best yield, which is similar to results published previously^[43]^. Use of larger filters likely results in loss of EVs in the filter^[44]^. Use of the smaller 10 kDa filter lead to an increase in protein which probably represents non-vesicular protein that was left in the later EV fractions and may decrease the EV purity. Using the 30 kDa filter to concentrate EV fractions yields the most EVs, while not introducing significantly increased extravesicular protein.

EE columns, while yielding the highest particle count and total protein, did so inconsistently and were not reproducible from sample to sample. In addition, there was a high amount of background protein in the BCA assay. This was further demonstrated by proteomic analysis, where EE preparations contained a large number of unique proteins. This may be an artifact of the affinity columns themselves (along with the increased particle count), or a result of non-specific binding of free protein or protein aggregate to the columns. UC also consistently yielded high particle counts as well as EV specific tetraspanins, but when visualized under transmission electron microscopy (TEM) (both when equivalent volume and when standardized to measured particle count), the SEC method had higher numbers of EVs as well as less background material that does not appear to be EVs. This background material likely explains the increase in total protein, tetraspanin staining on Western blot, and Nanosight counts in UC preparations. Given these findings, particle counts by Nanosight and tetraspanin antibody assays may overestimate the number of particles contained in EV samples isolated by UC^[12]^, especially in other biological samples that are not as dilute as BAL fluid.

Finally, certain EV isolation techniques may preferentially isolate some EVs, leading to differences in protein and nucleic acid composition of the final preparations. Interestingly, all three isolation methods studied yielded similar nucleic acid concentrations. This is likely because virtually all nucleic acids are found in protected EV environments. This is especially true in the lung, where RNAses and DNAses are prevalent to clear unwanted viscous nucleic acids^[45,46]^. In contrast, the proteins detected in the EV preps varied, which partially reflects varying levels of free protein contamination. EE had the highest amount of protein in EV preps, including many unique proteins not found in SEC or UC extracellular vesicle preparations. Pathway enrichment analysis suggested these excess proteins were from a different source than EVs, primarily reflecting intracellular processes found in cells undergoing stress and apoptosis. In contrast, gene ontology analysis of proteins found in all three EV preparations was highly enriched for immune and inflammatory processes, which would be expected based on the sources of BAL used in this study. Even lipoproteins, which are known to contaminate EV preparations^[19]^, were significantly more abundant in EE EV preparations.

There are limitations to our study. First, most of the BAL samples used were banked fluid from either patients undergoing clinically indicated bronchoscopy or from HIV-infected subjects participating in a study. While this does not diminish our isolation technique comparisons, since each BAL was processed using all three EV isolation methods, the proteomic analysis of the EVs did demonstrate an underlying inflammatory profile. This should not be interpreted as representative of BAL EVs from normal subjects. Furthermore, as inflammatory conditions are known to increase the generation of EVs^[47]^, EV yield from normal BAL may be lower than in our subjects. Finally, there are other EV isolation techniques we did not utilize in our study. While we speculated on the limitations of these other techniques, direct comparison with size exclusion chromatography was not performed. Thus, conclusions about the superiority of SEC over techniques not studied in this work should be tempered until direct comparisons are made.

**Figure 9:**
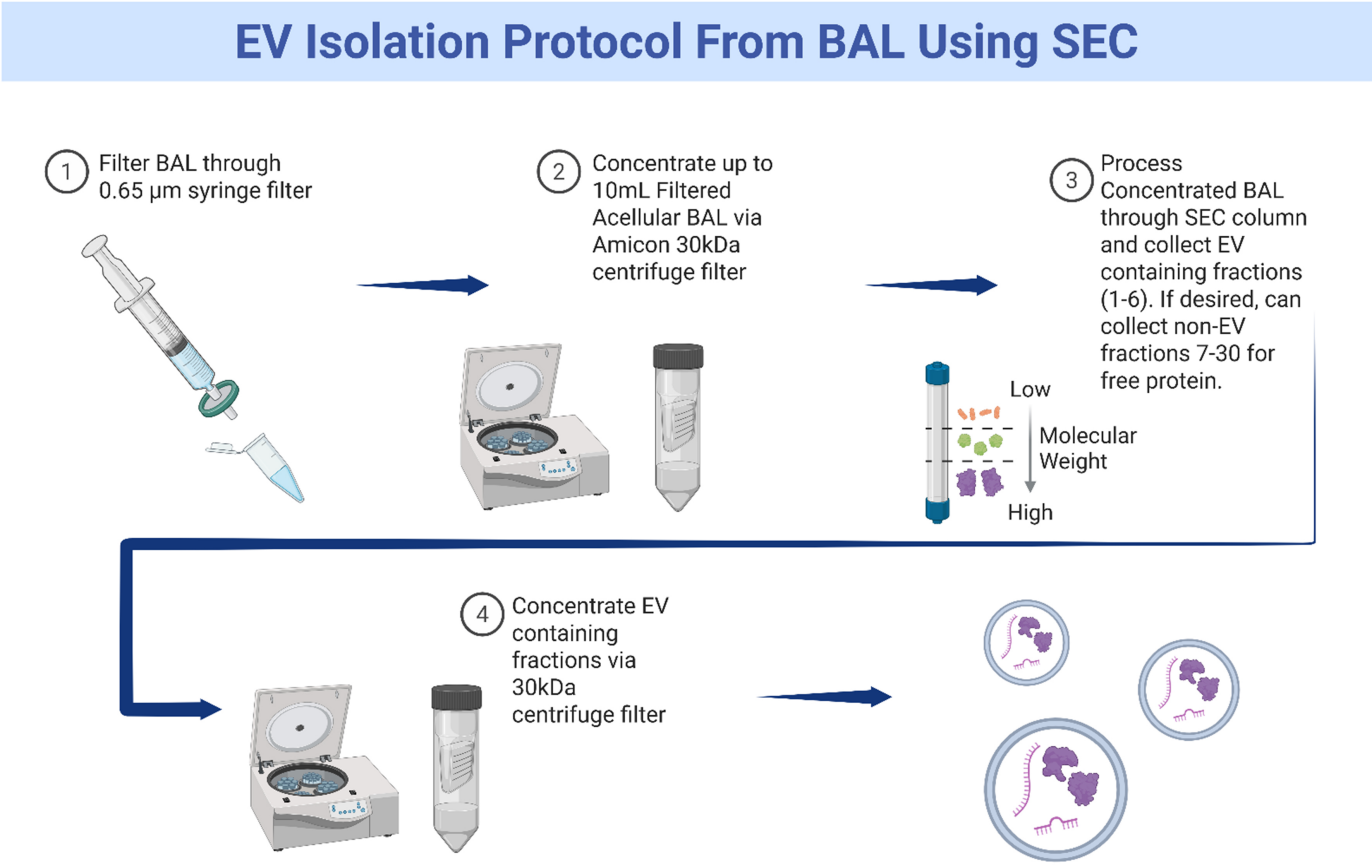
Graphical representation of method to isolate extracellular vesicles from bronchoalveolar lavage fluid. Created in BioRender. Fraser, M. (2026) https://BioRender.com/hjrs8dq. EV: Extracellular vesicle; BAL: bronchoalveolar lavage; SEC: size exclusion chromatography.

In conclusion, our study demonstrates that size exclusion chromatography is an excellent method to isolate EVs from bronchoalveolar lavage [Figure 9]. Samples prepared in this manner yield reproducible, high quantities of structurally intact, purified EVs with minimal extravesicular protein contamination.

## DECLARATIONS

### Availability of data and materials

Data is available from the senior author on request. In addition, per the Data Management and Sharing Plan for this project, data will be available in NHLBI BioData Catalyst.

### Copyright

© The Author(s) 2026.
