# Supplementary material for: Comparison of methods for isolation of extracellular vesicles from bronchoalveolar lavage fluid
Source: Extracell Vesicles Circ Nucl Acids. 2026 Apr 28;7(2):595–613. doi: 10.20517/evcna.2025.85 (PMC13174197; doi:10.20517/evcna.2025.85)
Supplement: Supplementary file 1 [file evcna-7-2-595-SupplementaryMaterials.pdf]

Supplementary Materials

Comparison of methods for isolation of extracellular vesicles from bronchoalveolar lavage fluid

Mark E. Fraser[1], Radha Patel[1], Taylor Shinabery[1], John Zagorski[1], Ling Chen[2], Navneet K. Dhillon[2], Matthias Clauss[1], Emma H. Doud[3,4,5], Amber L. Mosley[3,4,5], Benjamin Gaston[1], Homer L. Twigg III[1]

[1]Division of Pulmonary, Critical Care, Sleep, and Occupation Medicine, Indiana University Medical Center, Indianapolis, IN 46202, USA.
[2]Division of Pulmonary, Critical Care, and Sleep Medicine, The University of Kansas Medical Center, Kansas City, KS 66160, USA.
[3]Department of Biochemistry, Molecular Biology, and Pharmacology, Indiana University School of Medicine, Indianapolis, IN 46202, USA.
[4]Center for Proteome Analysis, Indiana University School of Medicine, Indianapolis, IN 46202, USA.
[5]Center for Computational Biology and Bioinformatics, Indiana University School of Medicine, Indianapolis, IN 46202, USA.

Correspondence to: Prof. Homer L. Twigg III, Division of Pulmonary, Critical Care, Sleep, and Occupation Medicine, Indiana University Medical Center, Indianapolis, IN 46202, USA. E-mail: htwig@iu.edu

Supplemental Table 1. Yield, inflammatory characteristics, and total protein concentrations of bronchoalveolar lavage fluid

	Clinical BAL lab specimens (*n* = 37)			HIV research subject specimens (*n* = 4)
	**Normal BAL differential (*n* = 8)**	**Lymphocytic predominant inflammation (*n* = 13)**	**Neutrophilic predominant inflammation (*n* = 16)**	
% Instilled Volume Returned (Median, IQ range)	37.0<br>33.0-41.5	42.0<br>27.0-49.3	33.0<br>13.5-43.5	61<br>47.3-64.3
% Alveolar Macrophages (Median, IQ range)	89.5<br>85.5-92.0	36.0<br>31.5-59.0	54.5<br>21.8-73.8	85.5<br>78.3-94.3
% Lymphocytes (Median, IQ range)	4.5<br>3.0-10.8	52.0<br>31.5-58.5	2.0<br>5.0-11.8	12.0<br>5.0-19.0
% Neutrophils (Median, IQ range)	4.0<br>2.0-7.0	6.0<br>2.5-8.5	38.0<br>19.8-70.8	1.0<br>0.3-2.5
% Eosinophils (Median, IQ range)	0.5<br>0.0-1.8	2.0<br>0.0-4.5	1.0<br>0.0-2.8	0.5<br>0.0-1.75
Total Protein in BAL (ug/ml) (Median, IQ range)	1,728<br>1,025-2,159	3,086<br>1,498-5,196	1,698<br>428-2,590	2,021<br>1,870-2,116

A

	Particle Count/mL (Nanosight)	Protein Concentration (BCA Assay)
Centrifuge + Filter	6.90E+09	25.56 ug/mL
Centrifuge alone	5.33E+09	48.22 ug/mL
Filter alone	3.97E+09	61.56 ug/mL

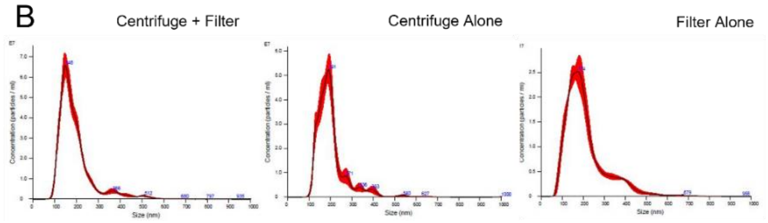

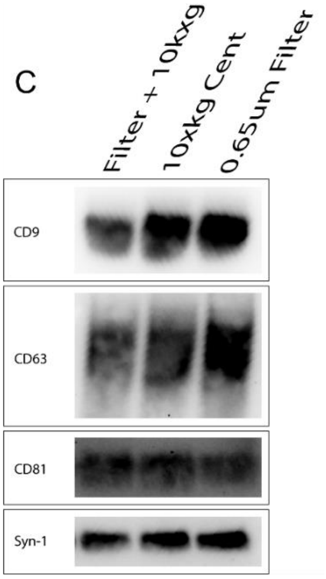

Supplemental Figure 1. Comparison of Centrifugation and 0.65 Micron Syringe Filter Prior to EV Isolation by SEC. BAL was clarified either by centrifugation at 10,000 × *g* for 40 min, filtration by 0.65 µm syringe filter, or a combination of the two methods prior to EV isolation by SEC to optimize yield and exclusion of large particles such as organelles and apoptotic bodies. (A) Table showing particle count by nanosight and total protein concentration by BCA assay with syringe filter demonstrating the highest total protein concentration and no significant difference between particle counts by nanosight; (B) Particle Size histograms from nanosight demonstrating equal distribution of particle sizes in all methods tested; (C) Western Blot of tetraspannins showing equal yield between centrifugation alone and filtration alone with a possible loss of some sample by CD9 and CD63 in the combined method, $n = 1$.

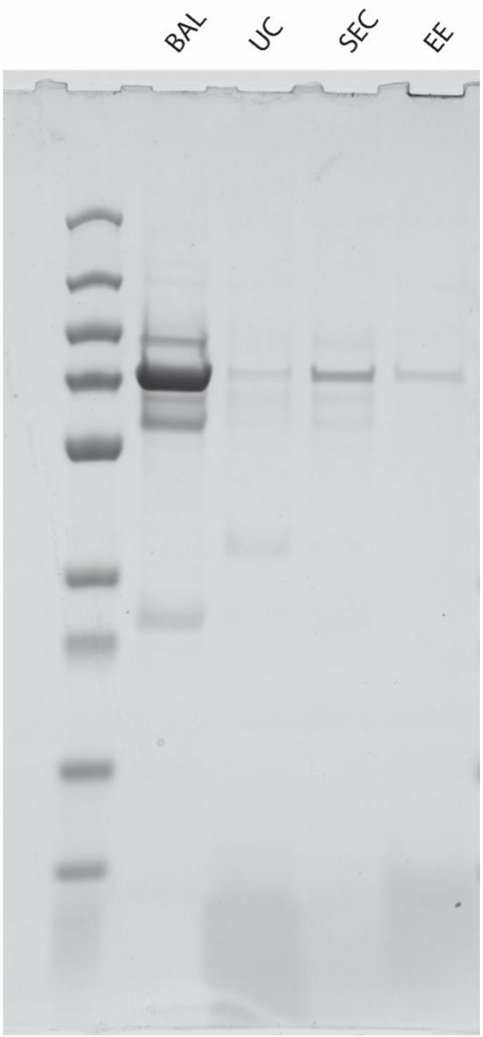

Supplemental Figure 2. Coomassie Blue staining of EV preparations. EV's isolated by size exclusion chromatography (SEC), ultracentrifugation (UC), and ExoEasy (EE) were run on a gel along with the neat BAL from which they were isolated. Coomassie Blue staining demonstrated that the bulk of free protein was removed from all three EV preparations.

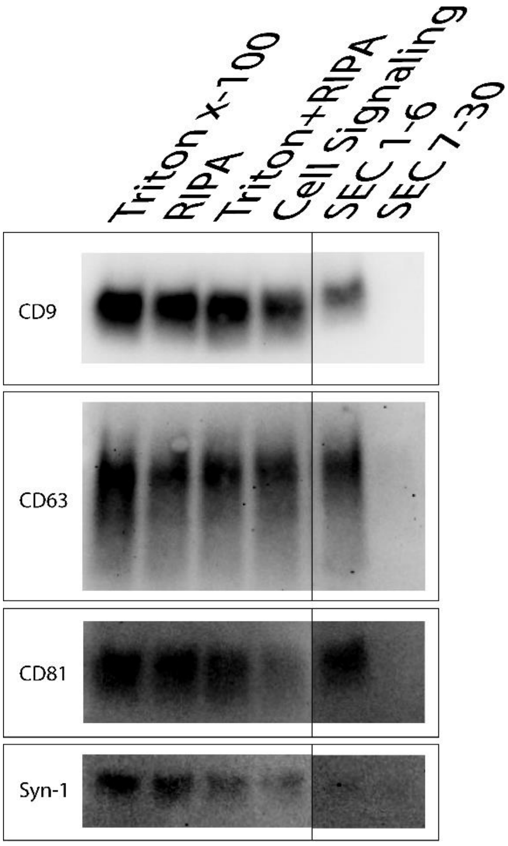

Supplemental Figure 3. Tetraspannin Detection by Western Blot After EV Lysis with Different Lysis Buffers. EVs isolated from acellular BAL by SEC were lysed with either Triton-× 100 0.1%, ThermoFisher RIPA Buffer, RIPA buffer with added Triton-x 100 0.1%, or Cell Lysis Buffer and compared to the control sample EVs (SEC 1-6) without EV lysis and free protein fractions (SEC 7-30), $n = 1$.
